# Expression and clinical significance of NLRC5 in hepatocellular carcinoma

**DOI:** 10.1080/15384047.2024.2390205

**Published:** 2024-08-12

**Authors:** Xiangyu Ma, Shangkun Ning, Tong Sun, Mei Liu, Jibing Liu

**Affiliations:** a Department of Interventional Surgical Oncology, Shandong Cancer Hospital and Institute, Shandong First Medical University and Shandong Academy of Medical Sciences, Jinan, Shandong, China; b Laboratory of Cell and Molecular Biology and State Key Laboratory of Molecular Oncology, National Cancer Center/National Clinical Research Center for Cancer/Cancer Hospital, Chinese Academy of Medical Sciences and Peking Union Medical College, Beijing, China

**Keywords:** HCC, NLRC5, tumor microenvironment (TME), tumor immune evasion, prognosis

## Abstract

NLRC5, the largest member of the nucleotide-binding and oligomerization domain (NOD)-like receptor (NLR) family, has been reported to participate in the regulation of immune function and is associated with chronic inflammatory diseases. However, the biological function of NLRC5 in hepatocellular carcinoma (HCC) has not been fully demonstrated. The aim of this study is to evaluate NLRC5 expression in the tumor tissues of HCC patients undergoing surgical treatment, assess its prognostic value, and explore its relationship with critical immune-related molecules within the tumor microenvironment. A total of 100 patients with hepatitis B virus-associated HCC receiving surgical treatment were enrolled in the study. Immunohistochemical results were obtained by scoring the intensity of cellular staining and the percentage of positive cells in the tissue sections. The association between NLRC5 expression levels and the main clinicopathological factors was analyzed by Chi-square test method. The prognostic values were analyzed by COX regression model and the Kaplan-Meier survival curve. Receiver operating characteristic (ROC) curve analysis was performed to assess the predictive performance of NLRC5 in postoperative patients with HCC. IHC showed that high expression of NLRC5 was observed in 67% of HCC tissue samples. Chi-square test showed that NLRC5 was a risk factor associated with tumor number, satellite nodule, and envelope invasion. Kaplan-Meier survival curves and COX survival analysis showed that high expression of NLRC5 was significantly associated with decreased overall survival (OS) in HCC patients (HR = 1.79, 95% CI 1.03–3.12, *p* = .041). However, univariate logistic regression analysis revealed that NLRC5 showed positive relationship with GZMB and CD8α suggesting its role in immune escape of HCC. ROC curve analysis showed that the combination of tumor number, envelope invasion, and NLRC5 expression (area under the curve = 0.824, sensitivity = 77.30%, specificity = 82.4%) can more accurately evaluate the prognosis of HCC patients compared to the combination of only tumor number and envelope invasion (area under the curve = 0.690, sensitivity = 43.9%, specificity = 94.1%).NLRC5 plays a crucial role in progression of HCC and can be considered as a potential prognostic and predictive biomarker. Targeting NLRC5 may provide an attractive therapeutic approach for HCC.

## 1. Introduction

Hepatocellular carcinoma (HCC) is one of the most common malignant tumors worldwide, with a consistently high mortality rate among cancer-related deaths.^1^ Currently, treatment options for patients with HCC include surgical resection, systemic therapy, radiotherapy, and chemotherapy. Surgical resection is still the major radical treatment method. Early diagnosis and treatment can significantly improve the prognosis and prolong the survival period of patients. Unfortunately, the majority of HCC patients are already in advanced stages when they first diagnosed, and most patients undergo tumor recurrence even metastasis after surgery, which results in poor prognosis.^2,3^ Previously, researchers have reported that the occurrence, progression, and recurrence of HCC are closely related to the functionality of the patient’s own immune system. HCC typically arises against a background of chronic inflammation and liver cirrhosis.^4^

The tumor microenvironment (TME) refers to the external environment in which tumor cells exist, composed of tumor cells, immune cells, fibroblasts, endothelial cells, cytokines, chemokines, and other components.^5^ An increasing amount of evidence suggests that certain protein molecules responsible for regulating immunity in the TME can significantly impact tumor growth and migration and are closely associated with clinical outcomes.^6^ Under the long-term influence of various factors such as viruses and alcohol, the liver undergoes oxidative stress response, resulting in the accumulation of free radicals such as reactive oxygen species and reactive nitrogen species, which cause varying degrees of damage to the liver. This process releases cell damage-associated molecular patterns and promotes the activation and recruitment of inflammatory cells, thereby altering the microenvironment of liver cells.^7^ Therefore, further research on the cellular characteristics of the liver TME and identification of TME-related biomarkers may provide new perspectives for the development and treatment of tumors .^8–11^

NLRC5 is the largest protein in the nucleotide-binding and oligomerization domain (NOD)-like receptor (NLR) family and serves as an important regulatory factor for nuclear factor kappa-B, type I interferon and inflammasome signaling pathways.^12^ The immune system can recognize cancer cells through tumor-specific antigens, but cancer cells can evade the immune system through various mechanisms.^13,14^ Impaired function of the major histocompatibility complex class I (MHC-class-I) antigen presentation pathway is one of the main mechanisms of tumor immune escape, and NLRC5 plays a crucial role in tumor immune escape by regulating the expression of MHC-class-I genes.^15,16^ NLRC5 can induce the expression of genes encoding key components through the MHC-class-I pathway, playing a critical role in tumor antigen presentation and recruitment/activation of CD8+ T cells.^17^ It was found that NLRC5-deficient mice exhibited a mild reduction in CD8+ T cells in peripheral lymphoid organs. The latest research indicates NLRC5 activation may be effectively augment cancer immunogenicity through MHC class I, highlighting NLRC5 as a cancer immunotherapy target.^18^ Kaplan-Meier survival analysis indicates a significant association between high NLRC5 expression and improved cumulative survival rates in melanoma, bladder, and cervical cancer.^19^ Additionally, NLRC5 plays an important regulatory role in mediating tumor inflammation response.^20^ Yo *et al*. found that NLRC5 is highly expressed in liver cancer and colorectal cancer, which may be associated with long-term stimulation of chronic inflammation.^19^ Despite the increasing number of reports on the role of NLRC5 in cancer, a literature gap regarding the expression and clinical significance of NLRC5 in HCC has not yet been explored, which warrants further investigation.

In the present study, we firstly examined the expression level of NLRC5 in tumor tissues of 100 HCC patients by immunohistochemistry, and analyzed its prognostic value. Then, based on our previous research,^21^ the correlation between NLRC5 and the expression of CD8α, FoxP3, CD56, CD68, CD31, pan-CK, and GZMB in HCC tissues were further analyzed, aiming to explore the potential role of NLRC5 in the TME of HCC.

## 2. Methods and materials

### 2.1. Patient selection

**Figure 1.**
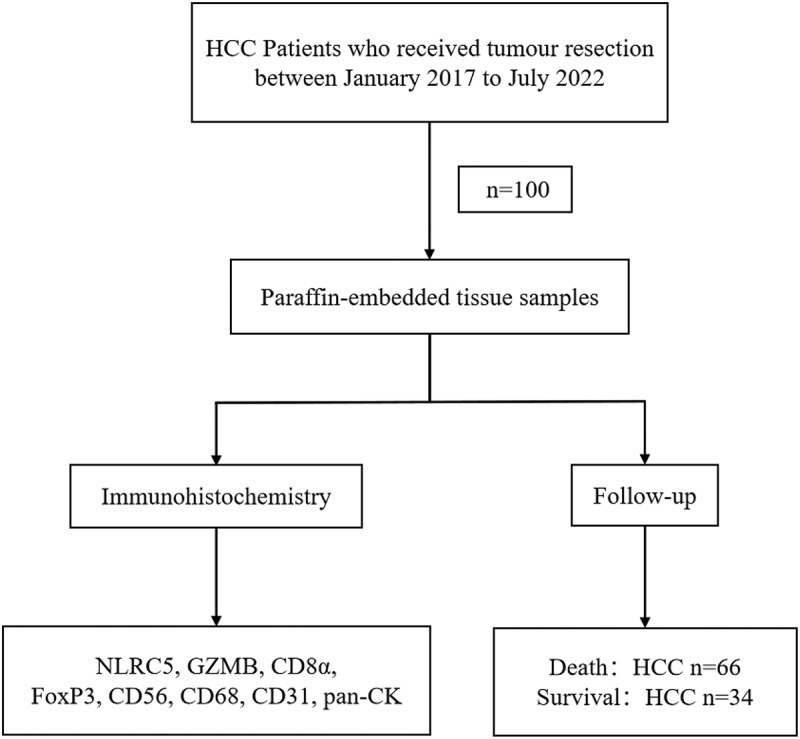
Flowchart of study design process.

A total of 100 HCC patients who underwent tumor resection at Shandong Cancer Hospital between January 2017 and July 2022 were included in this study. HCC diagnosis was confirmed according to the guidelines of the National Comprehensive Cancer Network (NCCN).^22^ We summarized the main clinical and pathological data of the patients, including microvascular invasion (MVI), tumor number, satellite nodule, tumor size, envelope invasion, and alpha-fetoprotein (AFP). Paraffin embedded HCC tissue samples were used for immunohistochemistry staining in this study. All clinical tissue specimens were stained with hematoxylin-eosin (HE) for identification in the Pathology Department. All patients were followed up until August 2023. The flowchart of the study design was shown in Figure 1. This study was approved by the Medical Ethics Committee of Shandong Cancer Hospital, and written informed consent was obtained from the participants.

All patients were followed up until death or August 1, 2023. HCC, hepatocellular carcinoma.

### 2.2. Immunohistochemistry and scoring

Tissue sections were cut at 4 µm. After deparaffinization and hydration, the tissue samples were subjected to antigen retrieval, and endogenous peroxidase activity was blocked by 3% hydrogen peroxide. The primary antibodies used were NLRC5 (diluted 1:200, orb94578; Biorbyt, Cambridge, UK) and GZMB (diluted 1:125, D6E9W; CST, MA, USA). Immunohistochemical staining was carried out using an automated immunohistochemistry staining machine (Leica BOND-MAX).

Scoring was performed based on the intensity of cellular staining and the percentage of positive cells.^21,23^ The presence of light yellow to brown color in tissue sections indicated positive cells. The staining intensity was scored according to the staining characteristics observed in the majority of cells (contrast to the background): no staining was scored as 0 (None), light yellow as 1 (Weak), brown yellow as 2 (Moderate), and brown as 3 (Strong). The percentage of positive cells was determined by the proportion of positive cells in a high-power field (200×): 0–10% was scored as 0, 11%-25% was scored as 1, 26%-50% was scored as 2, and > 50% was scored as 3. Five random 200× high-power fields were selected for each slide, and the staining intensity and positive cell count were scored for each field. The scores were added together to calculate the average: a total score of 0–2 was considered negative (denoted as “-”), 3–6 was considered positive (denoted as “+”). The immunohistochemical results were determined by two independent pathologists based on the staining scores. If the results are consistent, the outcome is adopted. In case of inconsistency, a reevaluation was performed until reaching consensus.

### 2.3. Follow-up

Postoperative follow-up was conducted through hospital-based follow-up visit and/or telephone inquiries. All patients were followed up until death or August 1, 2023. Routine postoperative follow-up included visits every 3 months for the first 2 years, and every 6 months starting from the third year. Each follow-up visit will include Alpha-fetoprotein (AFP), liver function, enhanced computed tomography (CT) scan, and/or digital subtraction angiography (DSA). If the above examinations raised suspicion of recurrence, enhanced magnetic resonance imaging (MRI) will be performed to confirm the diagnosis. The diagnosis of tumor recurrence was based on typical imaging findings and/or persistent elevation of serum AFP.

The treatment strategy for recurrence mainly involves comprehensive consideration of tumor characteristics, liver function, and general condition. Initially, local therapies are considered, including hepatic resection, radiofrequency ablation (RFA), transarterial chemoembolization (TACE), and radiotherapy. For advanced-stage recurrent HCC, systemic palliative therapies, such as immunotherapy, molecular targeted therapy, and chemotherapy are considered as alternative approaches for recurrent treatment. Overall survival (OS) is calculated based on the time from diagnosis to death or last follow-up.

### 2.4. Statistical analysis

Statistical analysis was performed using SPSS 26 and R software. Continuous data were expressed as mean ± standard deviation and compared using t-test (Student’s t-test). Ordered data were described as median within the interquartile range and compared using Wilcoxon rank-sum test. Categorical data were presented as numbers and compared using Chi-square test or Fisher’s exact probability test.

Assessing the association between NLRC5 and clinical parameters using Chi-square test. The correlation between NLRC5 or clinical parameters and overall survival of patients was analyzed by univariate and multivariate analysis in Cox regression model. P-values were calculated using the Wald test. Kaplan-Meier method was used to assess overall survival based on NLRC5 or clinical parameters. The p-value from Kaplan-Meier analysis was calculated using the log-rank test. Two-sided tests were conducted for p-values, and values less than 0.05 were defined as statistically significant. Receiver operating characteristic (ROC) curve and the area under the curve (AUC) were used to determine the predictive accuracy.

## 3. Results

### 3.1. Baseline characteristics of the study population

A total of 100 HCC patients were included in this study for analysis. The baseline characteristics of the patients are shown in Table 1. The average age of the patients was 57.03 ± 9.73 years, with 83% male. The majority of patients (93%) had liver cirrhosis in stages 1–2, and more than half of the patients (59%) had AFP levels <400 ng/ml. The maximum tumor size in these patients was 5.19 ± 3.41 cm, with the majority having a single lesion (69%) and no satellite nodule (85%). Approximately half of the patients had microvascular invasion (50%) and envelope invasion (45%). Most patients (85%) had liver function classified as Child-Pugh A. According to the Barcelona Clinic Liver Cancer (BCLC) Staging system, 81% of patients are classified as stage 0-A.

**Table 1.** Baseline characteristics of the hepatocellular carcinoma.

Characteristic	HCC(*n* = 100)
Age (years)^①^	57.03 ± 9.73
Gender, male/female.	83/17
Liver cirrhosis staging, 1-2/≥3	93/7
AFP (ng/ml), <400/≥400	59/41
Tumor size(cm)^①②^	5.19 ± 3.41
Tumor number, solitary/multiple.	69/31
MVI，absent/present.	50/50
Satellite nodule, absent/present.	85/15
Envelope invasion, absent/present.	55/45
Child-Pugh classification, A/B	85/15
BCLC staging, 0-A/B	81/19

Note.

① Data are presented as mean ± standard deviation.

② Represents the maximum diameter of the tumor.

HCC, hepatocellular carcinoma; AFP, alpha-fetoprotein; MVI, microvascular invasion; BCLC, Barcelona Clinic Liver Cancer.

### 3.2. The association between NLRC5 expression and clinicopathological factors in HCC patients

**Figure 2.**
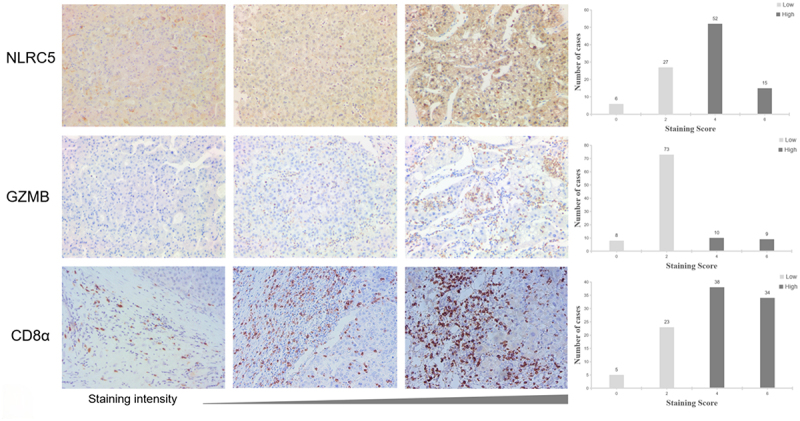
Immunohistochemical staining images.

We initially compared the expression of NLRC5 between HCC and adjacent non-tumor tissues through database analysis. The results revealed a significantly higher expression of NLRC5 in tumor tissues as compared to non-tumor tissues (Figure S1). Additionally, the expression of NLRC5 in 100 HCC tissue samples was examined by immunohistochemical methods. The results showed that NLRC5 was mainly expressed in the cytoplasm of the HCC cell, with 67% of HCC tissue samples showing high expression. As shown in Figure 2, it presents typical immunohistochemical staining images. The association between the expression level of NLRC5 and clinicopathological factors was evaluated using the Chi-square test. According to standard of staging system of liver cancer reported by NCCN and BCLC staging guidelines, we just analyzed the major clinicopathological factors, including AFP level (≥400 ng/ml), tumor size (≥5 cm), tumor number, MVI, satellite nodule, and envelope invasion, Child-Pugh classification and BCLC staging. As shown in Table 2, it was found that the expression level of NLRC5 was significantly associated with tumor number (*p* = .005), satellite nodule (*p* = .018), and envelope invasion (*p* = .001).

Representative immunohistochemical staining images of NLRC5，GZMB and CD8α expression. Distribution diagrams of staining intensity in 100 cases were shown, respectively. In HCC tissue samples, NLRC5 (67%) and CD8α (72%) primarily showed high expression, while GZMB (81%) showed low expression.

**Table 2.** Associations between the expression level of NLRC5 and clinicopathological factors.

Characteristic	NLRC5			
	n	Low expression	High expression	P value
		（N = 33）	（N = 67）	
AFP				
<400 ng/ml	59	20	39	
≥400 ng/ml	41	13	28	0.819
Tumor size				
<5 cm	65	20	45	
≥5 cm	35	13	22	0.656
Tumor number				
solitary	69	29	40	
multiple	31	4	27	**0.005**
MVI				
absent	50	19	31	
present	50	14	36	0.395
Satellite nodule				
absent	85	32	53	
present	15	1	14	**0.018**
Envelope invasion				
absent	55	26	29	
present	45	7	38	**0.001**
Child-Pugh classification				
A	85	28	57	
B	15	5	10	0.976
BCLC staging				
0-A	81	30	51	
B	19	3	16	0.076

AFP, alpha-fetoprotein; MVI, microvascular invasion; BCLC, Barcelona Clinic Liver Cancer.

### 3.3. The impact of NLRC5 and clinicopathological factors on patient survival

**Figure 3.**
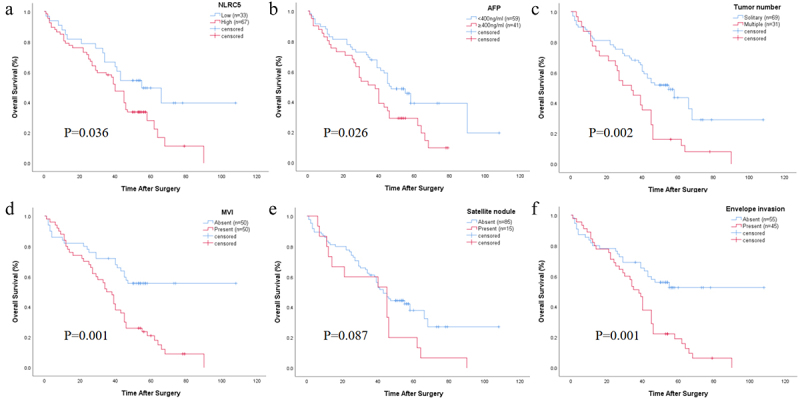
Kaplan-meier curves for OS in HCC patients.

To validate the impact of NLRC5 on HCC patient survival, we first performed prognostic analysis of NLRC5 in HCC tumor tissues and adjacent non-tumor tissues through the database. The results showed that the expression of NLRC5 was significantly associated with patient prognosis in tumor tissues, but not in adjacent non-tumor tissues (Figure S1). In our cohort, the overall survival (OS) rates of HCC patients at 1 year, 3 years, and 5 years were 81.0%, 61.0%, and 35.9%, respectively. The OS rates of NLRC5-positive patients at 1 year, 3 years, and 5 years were significantly lower than those of NLRC5-negative patients (79.1% vs. 84.8%, 58.2% vs. 66.7%, 28.1% vs. 49.6%). Survival analysis using Kaplan-Meier survival curves showed a significant negative correlation between NLRC5 expression and OS in HCC patients (*p* = .036), as shown in Figure 3. Additionally, AFP ≥400 ng/ml, multiple tumors, presence of MVI, envelope invasion, Child-Pugh B class and BCLC stage B were also significantly associated with decreased OS in HCC patients (*p* = .026, *p* = .002, *p* = .001, *p* = .001, *p* = .001, *p* = .001, respectively). Other clinical factors, including age, gender, stage of liver cirrhosis, tumor size, and satellite nodule, showed no statistically significant correlation with patient survival.

Kaplan-Meier curves were generated based on the expression levels of NLRC5 (A), AFP (B), tumor number (C), MVI (D), satellite nodule (E), and envelope invasion (F). AFP, alpha-fetoprotein; MVI, microvascular invasion.

The impact of NLRC5 and clinicopathological factors on the prognosis of HCC patients was investigated using univariate and multivariate COX survival analysis methods. Univariate analysis showed NLRC5 expression level (HR = 1.79,95% CI 1.03–3.12, *p* = .041), AFP (HR = 1.72,95% CI 1.06–2.81, *p* = .030), tumor number (HR = 2.15,95% CI 1.31–3.50, *p* = .002), MVI (HR = 2.37,95% CI 1.41–3.97, *p* = .001), envelope invasion (HR = 2.33, 95% CI 1.41–3.84, *p* = .001), Child-Pugh classification (HR = 0.13, 95% CI 0.07–0.26, *p* = .001), and BCLC staging (HR = 2.81, 95% CI 1.61–4.90, *p* = .001) were risk factors for OS in patients (Table 3). There was no significant correlation between other clinicopathological factors and patients’ OS. P-values less than 0.1 were included for further multivariate analysis. The results showed that tumor number (HR = 2.39, 95% CI 1.20–4.74, *p* = .013) and envelope invasion (HR = 2.09, 95% CI 1.13–3.89, *p* = .019) were independent risk factors affecting patients’ OS.

**Table 3.** Univariate and multivariate survival analysis of OS in 100 HCC patients.

Variable	Univariate analysis			Multivariate analysis		
	HR	95%CI	P value	HR	95%CI	P value
NLRC5	1.79	1.03-3.12	**0.041**			
Gender (male/female)	0.69	0.35-1.35	0.275			
Age (<60/≥60)	1.28	0.78-2.12	0.331			
Liver cirrhosis staging (1-2/≥3)	1.32	0.79-2.19	0.292			
AFP (<400/≥400)	1.72	1.06-2.81	**0.030**			
Tumor size(<5/≥5)	1.23	0.74-2.04	0.429			
Tumor number（solitary/multiple）	2.15	1.31-3.50	**0.002**	2.39	1.20-4.74	**0.013**
MVI (−/+)	2.37	1.41-3.97	**0.001**			
Satellite nodule (−/+)	1.65	0.92-2.95	0.094			
envelope invasion (−/+)	2.33	1.41-3.84	**0.001**	2.09	1.13-3.89	**0.019**
Child-Pugh classification (A/B)	0.13	0.07-0.26	**0.001**			
BCLC staging (0-A/B)	2.81	1.61-4.90	**0.001**			

HCC, hepatocellular carcinoma; HR, hazard ratio; CI, confidence interval; AFP, alpha-fetoprotein; MVI, microvascular invasion; BCLC, Barcelona Clinic Liver Cancer.

### 3.4. The predictive value of NLRC5 and clinicopathological factors on patient survival

**Figure 4.**
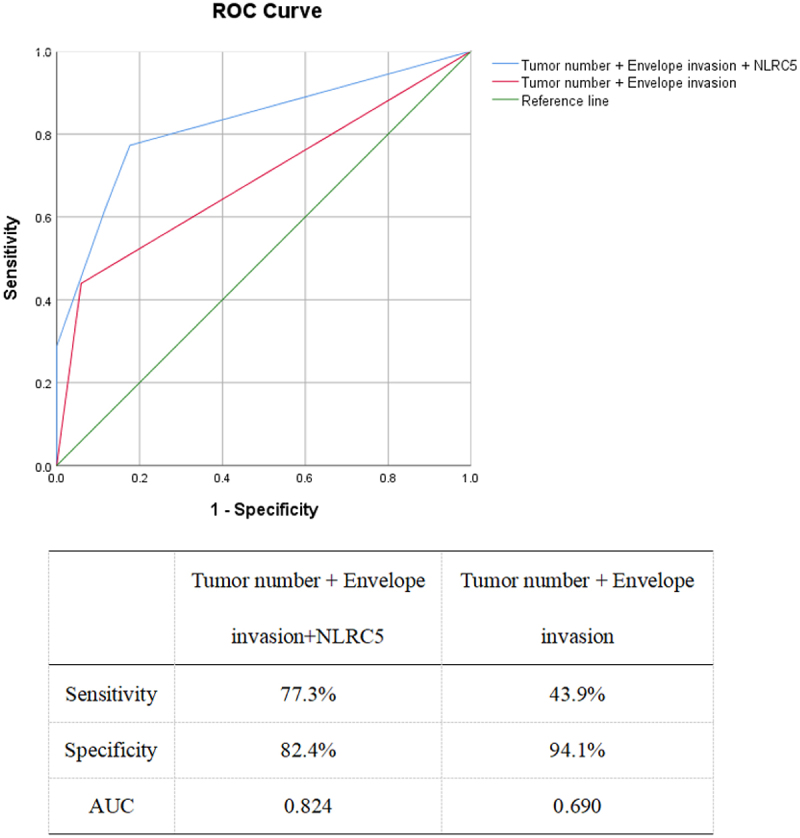
Receiver operating characteristic curve analysis for predicting the factors affecting patient survival.

We attempt to utilize the aforementioned identified high-risk factors to predict patients’ survival. Multivariate analysis revealed that tumor number and envelope invasion were independent risk factors affecting patients OS. The ROC curve was used to evaluate the ability of NLRC5 and clinicopathological factors to predict patient survival, and statistical analysis was performed on significant indicators. As shown in Figure 4, the ROC curve of the combination of clinicopathological factors (tumor number and envelope invasion) had an area under the curve (AUC) of 0.690 (sensitivity of 43.9%, specificity of 94.1%). The AUC for the combination of tumor number, envelope invasion, and NLRC5 was 0.824 (sensitivity 77.30%, specificity 82.4%). This suggests that the combination of clinicopathological factors and NLRC5 provides a more accurate prediction of survival in HCC patients.

The ROC curve model constructed by the combination of tumor number, envelope invasion, and NLRC5 is superior to the ROC curve model constructed by clinicopathological factors (tumor number and envelope invasion) (AUC: 0.824 > 0.690). Sensitivity, Specificity, and AUC are displayed below the ROC graph. ROC, receiver operating characteristic; AUC, area under the curve.

### 3.5. Correlation analysis between NLRC5 and CD8α, FoxP3, CD56, CD68, CD31, pan-ck, GZMB

Previously, we used immunohistochemistry to examine the expression of CD8α, FoxP3, CD56, CD68, CD31, and pan-CK in these 100 patients.^21^ Here, we further investigated whether there is a correlation between NLRC5 and the expression of CD8α, FoxP3, CD56, CD68, CD31, and pan-CK. The results of the univariate logistic analysis showed that the expression levels of NLRC5 and CD8α show a positive correlation (HR = 2.79, 95%CI 1.13–6.91, *p* = .027), as shown in Table 4. There was no significant association between NLRC5 and other molecular markers. Therefore, we further measured GZMB in these 100 patient tissue samples. GZMB, as an effector molecule of cytotoxic T cells, is closely related to the killing ability of CD8^+^ T cells. The results showed a significant correlation between NLRC5 and GZMB expression (HR = 3.96, 95% CI 1.08–14.53, *p* = .038). HCC tissues with high NLRC5 expression also demonstrate high expression of GZMB. Typical immunohistochemical staining images of GZMB and CD8α are shown in Figure 2.

**Table 4.** Univariate logistic analysis of NLRC5 and CD8α, FoxP3, CD56, CD68, CD31, pan-ck, GZMB.

Variable	NLRC5			
	HR	95%CI	P value	*β*
CD8α	2.79	1.13-6.91	**0.027**	1.026
FoxP3	0.85	0.36-1.99	0.711	−0.160
CD56	1.51	0.59-3.85	0.392	0.410
CD68	0.58	0.24-1.38	0.220	−0.544
CD31	1.31	0.57-3.03	0.524	0.272
pan-CK	1.11	0.42-2.96	0.836	0.104
GZMB	3.96	1.08-14.53	**0.038**	1.376

HR, hazard ratio; CI, confidence interval.

## 4. Discussion

In the present study, we assessed the expression of NLRC5 by using immunohistochemistry in HCC tumor tissues. The results indicated a significant correlation between the expression of NLRC5 and the OS of HCC patients. Patients with high expression of NLRC5 exhibited a significantly shorter OS compared to those with low expression of NLRC5 (*p* = .036). Furthermore, NLRC5 in combination with clinicopathological factors could provide a more accurate prediction of the survival period for HCC patients. This indicates that NLRC5 may have potential value as a prognostic molecular marker for predicting the survival and prognosis of HCC patients.

NLR is a protein family consisting of 22 members, all of which possess three structural domains and play important regulatory roles in mediating tumor inflammation response.^24^ Previous studies have shown that the activation of NLR is closely associated with various types of cancer, such as HCC, breast cancer and colorectal cancer.^25^ As the largest protein in the NLR family, NLRC5 plays a crucial role in innate immunity in eukaryotes. High expression of NLRC5 has been associated with favorable prognosis in many types of cancer. According to previous literature reports, patients with high expression of NLRC5 have significantly longer survival periods compared to those with low expression in melanoma, rectal, bladder, uterine, cervical, and head/neck cancer. Researchers believe that NLRC5 plays a key role in tumor MHC-class-I-dependent immune response and CD8^+^ T cell activation.^17^

In this study, we conducted immunohistochemical analysis on 100 cases of HCC tissue samples to demonstrate its role in HCC. The results showed that high expression of NLRC5 is a risk factor associated with poor prognosis in HCC patients, which differs from the performance of NLRC5 in other cancers. In cancers, such as melanoma, bladder, colorectal and cervical cancer, patients with high expression of NLRC5 have a better prognosis.^19^ In recent years, research has found that NLRC5 plays a crucial role in the regulation of immune function. Therefore, in order to clarify the role of NLRC5 in HCC, we further explored the correlation between NLRC5 and the expression of some immune cell markers and GZMB by univariate logistic analysis. GZMB is a cytotoxic granule enzyme with killing activity, which is secreted by cytotoxic T lymphocytes and natural killer cells.^17^ It is closely associated with the cytotoxic ability of CD8^+^ T cells. The results revealed a positive correlation between the expression levels of NLRC5 and CD8α, as well as GZMB, suggesting its role in immune escape of HCC. In addition to immune regulation, NLRC5 may also involve in the regulation of HCC malignant phenotype, which contributes to tumor progression. Previous studies have shown that NLRC5 was up-regulated in tumor tissues of HCC patients, and in vivo studies in mice have found that NLRC5 knock-down inhibited tumor growth by blocking the Wnt/β-catenin signaling pathway.^26^ Besides, NLRC5 can also regulate the expression of VEGF-A through the activation of the PI3K/AKT signaling pathway to mediate tumor angiogenesis and proliferation.^27^ At the same time, we also verified through the database that NLRC5 is associated with critical factors (such as CTNNB1 and VEGFA) related to the activity of these pathways (Figure S2). Additionally, NLRC5 has been found to promote activation and proliferation of hepatic stellate cells, leading to the formation of liver fibrosis.^28^ Therefore, we consider that NLRC5 is a risk factor associated with HCC, which may play roles in immune escape and HCC proliferation and angiogenesis. The current focus of HCC treatment research lies in PD-L1 immunotherapy. We have also found a significant correlation between NLRC5 and CD274 (Figure S2). Given the role of NLRC5 in cancer immune evasion, the efficacy of immunotherapy may be relatively poor for HCC patients with high expression of NLRC5. Inhibiting its expression, promoting its degradation and blocking its downstream pathways may be more meaningful in these HCC patients.

This study had some limitations, including its single-center design, a relatively homogeneous patient population and a lack of a validation dataset. A study of a multi-center large sample size is needed to strengthen generalizability of the findings. Furthermore, our study is a retrospective research analysis. In order to analyze the relationship between NLRC5 and these critical immune related molecules we have previously examined, we selected the same cohort.^21^ In future research, the validation of independent cohorts or functional studies are needed to strengthen the robustness of the findings.

In summary, our study found that NLRC5 is a risk factor associated with poor prognosis in HCC patients. The classifier established by the combination of NLRC5, tumor number, and envelope invasion were higher than that of just tumor number and envelope invasion. All these suggest that NLRC5 can be considered as a potential prognostic and predictive biomarker.

## Supplementary Material

Supplemental Material
