# Supplementary material for: Expression and clinical significance of NLRC5 in hepatocellular carcinoma
Source: Cancer Biol Ther. 2024 Aug 12;25(1):2390205. doi: 10.1080/15384047.2024.2390205 (PMC11321415; doi:10.1080/15384047.2024.2390205)
Supplement: Supplemental Material [file KCBT_A_2390205_SM2379.docx]

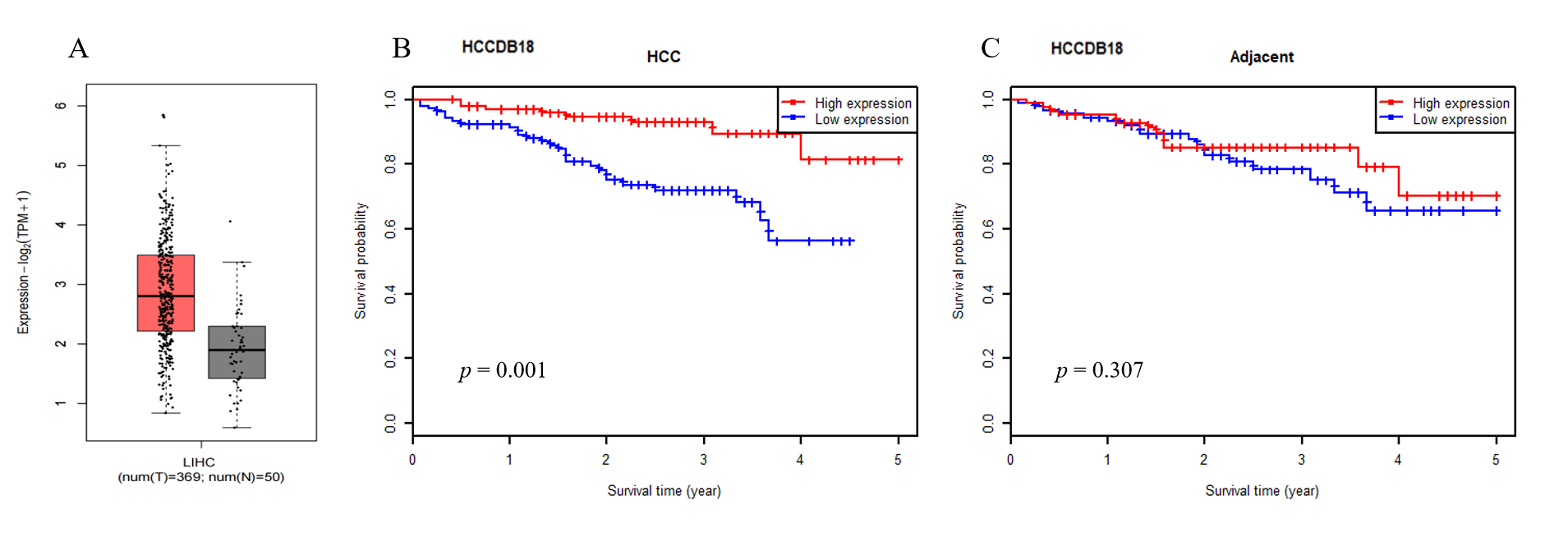


**Figure S1. Expression and prognostic analysis between HCC and adjacent non-tumor tissues based on the GEPIA database and HCCDB.**

The results of GEPIA^1^ database analysis showed that the expression of NLRC5 in tumor tissues was significantly higher than that in non-tumor tissues(A). The results of HCCDB^2^ analysis showed that high expression of NLRC5 in HCC tissues (B) was associated with a good prognosis of HCC patients, while in adjacent non-tumor tissues (C), NLRC5 expression had no significant correlation with the prognosis of HCC patients. HCC, hepatocellular carcinoma.

1. <http://gepia2.cancer-pku.cn/#correlation>
2. http://lifeome.net/database/hccdb/home.html


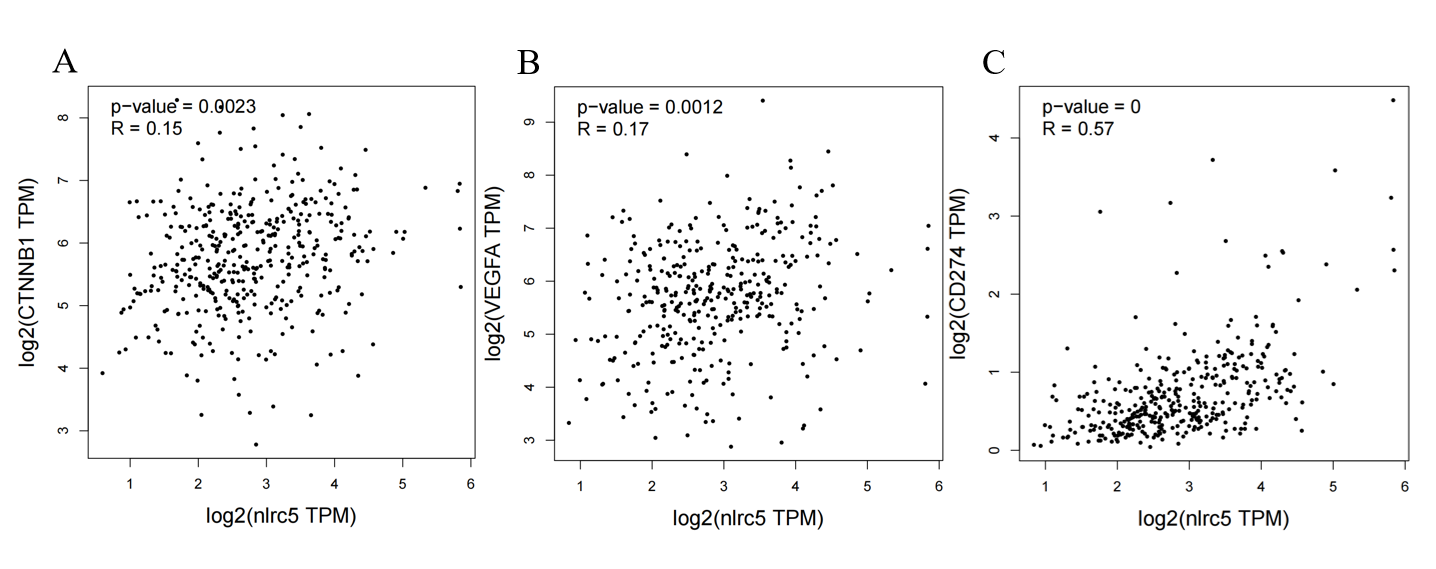
**Figure S2. The correlation analysis of NLRC5 with critical factors based on the GEPIA database.**

Scatter diagrams were generated using gene-related data from the GEPIA^1^ database. NLRC5 showed a clear correlation with CTNNB1 (A), VEGFA (B), and CD274 (C).

1. <http://gepia2.cancer-pku.cn/#correlation>
